# Identifying at-risk individuals for diseases of despair through integration of clinical practice and social service systems

**DOI:** 10.1017/cts.2024.548

**Published:** 2024-05-22

**Authors:** William A. Calo, Chelsea M. Bufalini, Katherine Spanos, Michele Sandoe, Cinda Watkins, Jordan Lewis, Gail D’Souza, Jamelia Graham, Josheili Llavona-Ortiz, Deepa Sekhar

**Affiliations:** 1 Department of Public Health Sciences, Penn State College of Medicine, Hershey, PA, USA; 2 Penn State Clinical and Transitional Science Institute, Hershey, PA, USA; 3 Unite Us, Pittsburgh, PA, USA; 4 United Way of Southwestern Pennsylvania, Pittsburgh, PA, USA; 5 Pennsylvania Department of Drug and Alcohol Programs, Harrisburg, PA, USA; 6 Department of Pediatrics, Penn State College of Medicine, Hershey, PA, USA

**Keywords:** Diseases of despair, suicide, substance use, helpline, rural populations

## Abstract

**Background:**

Individuals who are unable to meet their basic needs are more likely to respond reactively to their immediate social and financial hardships with behaviors that lead to “diseases of despair,” which include suicide, drug overdose, and alcohol-induced liver diseases. We sought to assess the feasibility of a community-to-clinic referral approach for diseases of despair-related behaviors.

**Methods:**

Guided by the Model for Adaptation Design and Impact, we adapted existing clinical risk assessments into a six-item screener and integrated it into the PA 211 Southwest helpline’s workflow. The screener was created to identify helpline callers at risk for suicidal ideation/behavior, alcohol abuse, drug use, and those in need of seasonal flu vaccination. The screener was implemented from December 2020 to March 2021. We invited at-risk individuals who accepted a service referral to complete baseline and follow-up surveys to learn about their satisfaction with screening and use of referrals.

**Results:**

2,868 callers were invited to take the screener, with 37% (*n* = 1047) participation. Among screened callers, 19% (*n* = 196) were at risk of alcohol abuse, 11% (*n* = 118) for drug use, 9% (*n* = 98) for suicidal ideation/behavior, and 54% (*n* = 568) needed flu vaccination. Of those, 265 callers accepted at least one of the offered referrals. Forty-seven individuals took our surveys, with almost half of them (*n* = 22) reported engaging with a referral and 90% recommended the helpline for health referrals.

**Conclusion:**

Our findings demonstrate the feasibility of using existing community infrastructure and social service systems to actively screen and link at-risk individuals to needed health referrals in their communities.

## Introduction

Americans are suffering a decline in life expectancy, a public health reversal that has not been observed in any other industrialized country [1]. Recent data suggests this trend is driven by outsized increases in mortality rates due to suicide, drug overdose, and alcohol-induced liver diseases, a phenomenon called “diseases of despair” [2,3]. Rates of diseases of despair have increased nationally by 50% from 2005 to 2017, but even more rapidly in Pennsylvania, by 96% over the same period [4]. At the population level, these increases in premature mortality have coincided with decades of economic decline for less educated and unskilled workers [5]. Researchers have conceptualized a pathway in which despair due to economic stagnation and financial stressors leads to behaviors that increase the risk of death by suicide, drug overdose, and alcoholic liver disease [6]. Data supports this pathway, with those at the highest risk of deaths from diseases of despair including unemployed adults and people with net income losses [7]. Existing evidence also suggests that financial losses especially among those with low education levels predict an increase in harmful despair-associated behaviors that increase mortality [8].

Despite the increasingly high prevalence of mental health and substance use problems in the USA, many adults do not receive treatment due to undiagnosed disorders [9,10]. Regular screenings in healthcare settings enable early identification of these life-threatening behaviors, which translate to early care [10,11]. However, there are still significant barriers to these screenings, including low awareness among clinic staff and high levels of stigma among patients [12,13]. Not only this, but the individuals most severely affected by diseases of despair are unlikely to even make it to the medical office for potential screening and referrals for treatment [14– 16]. Therefore, it is critical to find ways to engage at-risk individuals for diseases of despair-related behaviors outside of the typical healthcare setting to connect them to needed behavioral health programs in their communities.

The United Way 211 helpline is uniquely positioned to reach individuals at risk for diseases of despair. The 211 helpline is a federally designated dialing code part of a national initiative to connect callers with local health and social service agencies that aid in meeting basic needs (e.g., utility payments, food provision) [17]. Callers speak with trained resource navigators who identify the caller’s needs, search a database to find local resources, and provide referrals to help callers contact service providers [17]. The 211 helpline has been used to connect callers with underutilized health services such as smoking cessation programs [18,19], vaccination [20], and cancer screening [21,22]. Despite the helpline reach, utilization of the 211 to screen callers for diseases of despair-related behaviors and connection with needed health referrals has not previously been evaluated.

We sought to fill this gap by exploring the feasibility of a community-to-clinic referral approach for diseases of despair. We partnered with the PA 211 Southwest (PA211SW) helpline on a novel intervention to adapt a set of clinical risk assessment tools for use with diseases of despair-related behaviors to screen and refer callers to needed health services. The PA211SW catchment area includes Appalachian counties that are disproportionately affected by mortality due to diseases of despair; death rates are 37% higher in the Appalachia than in the non-Appalachia region [4,23]. Additionally, in recognition of the connection between diseases of despair and limited preventive care access, we also considered opportunities to refer individuals to flu vaccination services. If successful, this type of community-driven intervention can be disseminated and implemented nationwide through the large network of 211 helplines operating across the USA.

## Methods

### Participants

On average, the PA211SW receives about 4600 calls every month from community members seeking assistance to help meet a basic need throughout their 13-county catchment area in Southwestern Pennsylvania. Potential participants included those contacting the PA211SW and conversing with one of their helpline’s resource navigators. We trained 16 resource navigators in research ethics and conducted behavioral rehearsals [24] to mimic the call flow and recruitment procedures prior to beginning recruitment. Upon initiating recruitment, we also incorporated quality checks by listening to a sample of calls to analyze resource navigator fidelity in the recruitment protocols and offered any required improvements. Data reports of recruited participants were exported daily (Monday–Friday) from the United Way’s 211 RTM system to the research team at Penn State College of Medicine to invite potential participants to survey activities. All recruitment activities occurred from December 2020 to March 2021. The Institutional Review Board of the Pennsylvania State University reviewed and approved the study.

### Intervention procedures

We adapted existing clinical risk assessments [25,26] to a six-item screener to be used by the nonclinical resource navigators at the helpline. This screener went through numerous rounds of revisions from all collaborators, including incorporating feedback from the resource navigators themselves after the behavioral rehearsals. The finalized screener was implemented into the existing workflow of PA211SW with both the resource navigators at the call center and online on their website. Resource navigators followed up with those who completed our screener on the PA211SW website.

With each call, resource navigators would determine that a caller was not in immediate distress and provide the caller with referrals for their presented need before inviting them to participate in the screener. The risk assessment screener was introduced to callers as a way to assist them with additional health referrals and as completely voluntary and confidential. For those who agreed to be screened, the resource navigators verbally administered the 6-item screener over the phone. Callers were asked about behaviors related to diseases of despair (see Measures). Additionally, callers were asked if they received a flu shot in the last six months. Resource navigators followed prompts to determine if a caller was at risk for one or more diseases of despair-related behaviors or needed the seasonal flu vaccination (Supplementary file 1).

Based on the caller’s responses, they were offered referrals to local services as needed. Callers who accepted some or all of the referrals were provided them according to the normal workflow of the PA211SW and were then invited to participate in our surveys to evaluate their experience with the screening and referral processes. Those that agreed to take the surveys and provided a valid email address were contacted by the research team within three days via an email invite to REDCap, a secure web-based application that supports data capture for research studies [27]. Participants completed a baseline survey and received a follow-up survey 30 days later. Our intervention protocol was guided by the Model for Adaptation Design and Impact as it provided a rubric for deliberating and documenting the different adaptations for this project that took place, why those adaptations took place, and which outcomes the adaptations were designed to improve [28].

### Measures

#### Risk assessment screener

The screener first assessed alcohol use with one question: “In the past year, how many times have you had (4 for women/5 for men) drinks such as a glass of wine, a can of beer, or a shot of liquor in one day?” The screener also asked two questions about drug use in the past year, one about using “prescription drugs for nonmedical reasons” and the other about using “drugs such as nonmedical marijuana, cocaine, heroin or other recreational drugs.” All questions asking about alcohol or drug use had response options of never, once, or twice in the past year, monthly, weekly, daily, or almost daily, and the caller preferred not to answer. Those who selected monthly, weekly, or daily or almost daily screened at risk for alcohol or drug abuse. The screener asked about both suicidal ideations and attempts in the last six months with questions about having “thoughts of ending your life” and if “you attempted to end your life” (yes/no). If callers answered “yes” to either question, they were considered at risk for suicidal behaviors. Lastly, the screener assessed the need for a flu shot with one question, “In the past six months, have you had your flu shot or influenza vaccine?” (yes/no). Callers identified at risk for diseases of despair-related behaviors or in need of flu vaccination were offered corresponding health referrals and the opportunity to participate in our survey study.

#### Intentions and referral engagement

The baseline survey gauged participants’ intent to seek the referral (i.e., plan to seek the referral, do not plan to seek the referral, unsure), while the follow-up survey measured their actual engagement with the referral. Engagement was defined as any of the following: searched for more information about the referral, called and spoke with someone, scheduled an appointment, or agreed to be directly transferred by the resource navigator to the referral (when this option was available). If more than one referral was provided, these questions were asked separately for each referral.

#### Satisfaction

In the baseline survey, participants were asked to assess their satisfaction with resource navigators and helpline. They were asked to rate their level of agreement with statements saying that the resource navigator was “easy to interact with,” “trustworthy,” “knowledgeable,” “respectful,” and “helpful.” The final statement was, “I would recommend others call the 211 to get referrals for health services.” All of these were assessed on a five-point Likert scale.

#### Demographic variables

Demographic information provided by the PA211SW database included the caller’s county, age, sex, veteran status, and whether they have children in household (Table 1). Additional demographic characteristics were assessed in the baseline survey, including race and ethnicity, sex, employment status, and healthcare information. See Supplementary file 2 for our survey instrument.

**Table 1.** Characteristics of callers who were invited to participate in screener (*n* = 2868) and survey participants (*n* = 47)

Characteristics	*N* (%)
*Helpline callers who took screener (n = 2868)*	
*County of residence*	
Allegheny	1842 (64)
Westmoreland	154 (5)
Beaver	77 (3)
Butler	71 (2)
Washington	58 (2)
Mercer	55 (2)
Other	611 (21)
*Age, mean (SD)*	46.8 (15.8)
*Sex*	
Female	1984 (69)
Male	750 (26)
Other or not reported	134 (5)
*Veteran*	175 (6)
*Children in household*	825 (29)
*Survey participants (n = 47)*	
*Age, mean (SD)*	39.6 (12.3)
*Race/ethnicity*	
Non-Hispanic White	27 (57)
Non-Hispanic Black	17 (36)
Hispanic	1 (2)
Other	2 (4)
*Sex*	
Female	35 (75)
Male	12 (25)
*Unemployed*	27 (59)
*Household income*	
$15,000 or less	28 (60)
$15,001 or more	19 (40)
*Confidence to take care of own’s health*	
Completely/very	26 (57)
Somewhat/a little/not at all	21 (43)
*Do not have a doctor*	19 (40)

### Data analysis

Screening and referral data were recorded by resource navigators and shared with the research team. Screening, referrals, and survey data were analyzed and reported using descriptive statistics. Given the short term of this project, we were unable to assess changes in rates of diseases of despair-related behaviors, which is our long-term goal. Instead, we sought to estimate the proportion of eligible callers who may be at risk of diseases of despair-related behaviors or those who needed flu vaccination. We used the chi-squared test or Fisher’s exact test (to account for multiple cell counts < 5) to determine statistically significant differences between screener results and referrals provided by key demographic variables (sex, age group, veteran status, and children in household). While the chi-squared test relies on an approximation assuming the sample is large, Fisher’s exact test is one of exact tests, which is an adequate statistical method when dealing with small samples [29]. Accordingly, chi-squared tests were used and reported except when the cell counts were < 5; in those cases, we used the Fisher’s exact test. We reported the *p* value for the corresponding test. Additionally, for those callers who completed our survey, we assessed callers’ engagement with referrals and their satisfaction with screening and referral processes. This information is essential in understanding the need and opportunity for intervention programming through 211 helplines and provides a basis for estimating sample size and power calculations for future trials. For reporting, survey questions that were originally asked with a five-point Likert scale were dichotomized to “Disagree/Neither” and “Agree.” All analyses were performed using SAS version 9.3.

## Results

A total of 2868 callers were invited to complete the screener, and 37% (*n* = 1047) agreed to participate. Among screened callers, 19% (*n* = 196) were at risk for alcohol use disorder, 11% (*n* = 118) for drug use disorder, and 9% (*n* = 98) for suicidal ideation or behavior (Table 2). Additionally, 54% (*n* = 568) of screened callers needed the flu vaccine. In total, 690 of callers (66% of those screened) were eligible for a referral based on their screener responses, with 265 of them (25% of those screened) accepting at least one of the offered referrals. Resource navigators provided referrals for substance use (alcohol or drug) to 110 callers, referrals for suicidal behaviors to 48 callers, and referrals for flu vaccination services to 122 callers. Forty-seven callers who received a referral agreed to participate in the baseline survey, and of those, 35 (74%) participants completed the 30-day follow-up survey.

**Table 2.** Screening results and referrals provided

	*N* (%)
*At-risk behaviors based on screener* ^*1*^	
Alcohol abuse	196 (19)
Drug abuse	118 (11)
Suicidal ideation	98 (9)
Flu shot	568 (54)
None	359 (34)
*Referrals provided to callers* ^*2*^	
Alcohol and/or drug use	110 (16)
Suicidal thoughts or attempt	48 (7)
Flu vaccine	122 (18)
Caller refused referral	425 (61)

*Note:* ^1^ *N* = 1047; callers could be identified as being at risk for more than one health issue. ^2^ *N* = 690; callers could be referred to more than one service.

Screening at risk for drug abuse and suicidal thoughts was significantly higher among callers not reporting their gender (18%, respectively) (Table 3). By age group, callers between the ages of 18 and 39 years old had the highest number of at-risk screens for drug abuse (15%; *p* < 0.01). Callers who opted out of reporting their veteran status had a significantly higher number of screenings for suicidal thoughts (16%; *p* < 0.05). We found a higher number of callers needing the flu vaccine among those between the ages of 18 to 39 years old (67%; *p* < 0.01), not being veterans (57%; *p* < 0.01), and reporting having children in their household (64%; *p* < 0.01). Regarding the types of referrals offered to callers (Table 4), the highest proportions of respondents receiving a referral by type and demographic characteristics were mostly those who did not disclose their gender, age group, if there were children in their household, or their veteran status. On the other hand, most women (74%; *p* < 0.01), people 65 years or older (86%; *p* < 0.01), those with no children (74%; *p* < 0.01), and veterans (87%; *p* < 0.01) refused the referrals provided.

**Table 3.** Associations between screening results and demographic characteristics (*n* = 1047)

	Alcohol abuse n/N (%)	Drug use n/N (%)	Suicidal thoughts or attempt n/N (%)	Need flu vaccination n/N (%)
Gender				
Male	56/264 (21)	40/264 (15)**	22/264 (8)*	149/264 (56)
Female	124/703 (18)	64/703 (9)	62/703 (9)	378/703 (54)
Other/not reported	16/80 (20)	14/80 (18)	14/80 (18)	41/80 (51)
Age group				
18–39	71/332 (21)	50/332 (15)**	30/332 (9)	223/332 (67)**
40–64	88/450 (20)	48/450 (11)	46/450 (10)	235/450 (52)
>65	11/110 (10)	4/110 (4)	5/110 (5)	33/110 (30)
Not reported	26/155 (17)	16/155 (10)	17/155 (11)	77/155 (50)
Children				
No	97/453 (21)	50/453 (11)	49/453 (11)	237/453 (52)**
Yes	58/328 (18)	43/328 (13)	23/328 (7)	210/328 (64)
Not reported	41/266 (15)	25/266 (9)	26/266 (10)	121/266 (45)
Veteran status				
No	163/845 (19)	99/845 (12)	74/845 (9)*	483/845 (57)**
Yes	6/62 (10)	4/62 (6)	2/62 (3)	16/62 (26)
Not reported	27/140 (19)	15/140 (11)	22/140 (16)	69/140 (49)

*Note:* Callers could be identified as being at risk for more than one health issue. Chi-squared tests were used except when the cell counts were < 5; in those cases, we used the Fisher’s exact test. *p < 0.05 and **p < 0.01 for the corresponding test.

**Table 4.** Associations between referral type received and demographic characteristics (*n* = 690)

	Alcohol abuse and/or drug use n/N (%)	Suicidal thoughts or attempt n/N (%)	Flu vaccination n/N (%)	Caller refused referral(s) n/N (%)
Gender				
Male	30/179 (17)**	8/179 (4)**	28/179 (16)**	126/179 (70)**
Female	58/459 (13)	26/459 (6)	57/459 (12)	339/459 (74)
Other/not reported	21/52 (40)	14/52 (27)	34/52 (65)	5/52 (10)
Age group				
18–39	43/260 (17)*	16/260 (6)**	34/260 (13)**	184/260 (71)**
40–64	40/292 (14)	18/292 (6)	38/292 (13)	213/292 (73)
≥65	2/43 (5)	0/43 (0)	4/43 (9)	37/43 (86)
Not reported	24/95 (25)	14/95 (15)	43/95 (45)	36/95 (38)
Children				
No	41/299 (14)	18/299 (6)*	37/299 (12)**	222/299 (74)**
Yes	35/239 (15)	12/239 (5)	28/239 (12)	173/239 (72)
Not reported	33/152 (22)	18/152 (12)	54/152 (36)	75/152 (49)
Veteran status				
No	80/575 (14)**	32/575 (6)	77/575 (13)**	417/575 (73)**
Yes	2/23 (9)	0/23 (0)	1/23 (4)	20/23 (87)
Not reported	27/92 (29)	16/92 (17)	41/92 (45)	33/92 (36)

*Note:* Callers could receive more than one referral. Chi-squared tests were used except when the cell counts were < 5; in those cases, we used the Fisher’s exact test. *p < 0.05 and **p < 0.01 for the corresponding test.

In the baseline survey, a quarter of those (5/19; 26%) who received a substance abuse referral had plans to seek it within the next month, while the other 74% were unsure (*n* = 8) or did not plan to seek the referral (*n* = 6). For the suicide prevention referral, over one-third of participants (5/13; 38%) were planning to seek it, while the rest were either unsure (*n* = 6) or did not plan to seek it (*n* = 1). Based on the experience of resource navigators in crisis response, one caller was immediately connected to a mental health crisis service. Forty percent (10/25) of survey participants who received a referral for flu vaccination were planning to seek it, with the other callers reporting being unsure (*n* = 12) or not planning to seek it (*n* = 3). In the follow-up survey, of those referred for alcohol or drug use services, 60% (12/20) reported engagement with the referral. Of those referred for suicide prevention or crisis services, over two-thirds (9/13) engaged with the referral. Less than one-fourth (4/13) of individuals who received a referral for flu vaccination engaged with it.

Participants had overall positive perceptions of the resource navigators (Table 5), most agreeing that they were trustworthy (*n* = 38; 81%), knowledgeable (*n* = 41; 87%), helpful (*n* = 43; 91%), easy to interact with (*n* = 41; 87%), and respectful *(n* = 42; 89%). The majority of participants (*n* = 42; 89%) also reported high satisfaction with the screening and referral process by agreeing they would recommend the helpline for health referrals to other people.

**Table 5.** Satisfaction with resource navigators and helpline (*n* = 47)

	*N* (%)
*The resource navigator was…*	
Easy to interact with	41 (87)
Trustworthy	38 (81)
Knowledgeable	41 (87)
Respectful	42 (89)
Helpful	43 (91)
*Recommend others call the 211 to get referrals for health services*	42 (89)

*Note:* Percentages indicate survey participants who responded “somewhat agree” or “strongly agree.”

## Discussion

The present study demonstrates the feasibility of using existing community infrastructure and social service systems, like the PA211SW, to actively screen and link at-risk individuals to needed health referrals in the communities they live. First, 37% of callers who were invited to take the screener accepted it, suggesting that using the 211 helpline to conduct preventive health screeners is an acceptable offering for their clients even if they were calling for other purposes. In a randomized controlled trial with adults served by the Missouri 211 helpline, Kreuter et al. invited a random sample of callers to complete a cancer risk assessment to receive up to three health referrals for needed services (e.g., mammography, pap testing, colonoscopy, human papillomavirus vaccination, and smoking cessation) [30]. Forty-eight percent of callers who were invited to take the risk assessment completed it. It is important to note that the demographic characteristics of the callers in our study are different from those reported by Kreuter et al. and that we conducted a screener for health behaviors with high levels of stigma among the public. Second, although the screener was not designed to diagnose any medical condition, it helped to identify individuals who may benefit from health services’ referrals as showed by the 25% of screened callers who accepted at least one of the referrals made by the resource navigators. Lastly, our survey data showed that many of these callers engaged with the provided referral. Future research should explore what factors motivate or facilitate people’s engagement with health referrals and how the referrals made by a helpline improve health outcomes associated with diseases of despairs.

Our feasibility data suggests that callers think very highly of resource navigators and trust their health recommendations. Standard training for resource navigators includes crisis intervention, mental illness and special needs populations, computer system software, telephone system, referral database, and documentation requirements. They are also certified by the Alliance of Information and Referral Systems (AIRS). This level of capacity ensures that resource navigators respond to callers’ inquiries and helpline’s initiatives according to established service and quality standards, and our study was no exception. In our behavioral rehearsals and quality checks, we observed their empathy, patience, and professionalism when referring callers to the appropriate agencies that have the necessary resources to assist them with their basic needs and health services. All this is reflected in the positive scores that survey participants gave when rating their experience with these resource navigators.

Future research is needed to continue advancing the development and scalability of interventions, like ours, that leverage existing community infrastructure to address the unmet social needs of individuals in communities experiencing high rates of diseases of despair. For example, experimental studies should examine the impact of various modalities of community-to-clinic referrals on treatment initiation and reduced rates of substance use, alcohol abuse, and suicide. Studies should also evaluate the mechanisms of how the utilization of services to address social determinants of health (e.g., food insecurity, housing instability, economic circumstances, safety) improves diseases of despair-related behaviors. There is also a need for studies that utilize implementation science to test strategies for overcoming barriers to the adoption, adaptation, and integration of social needs programs in clinical settings.

Our study has several strengths. First, we chose to pilot our community-to-clinic referral approach within the 211 system. Thus, the implementation locale was representative of real-world community-based settings and has broad reach into populations who are disproportionately poor and unemployed, those at higher risk of diseases of despair. Second, the intervention was delivered by 211 resource navigators who had proper training in how to manage confidential information and deal with diverse populations and situations. Leveraging on the existing workforce in other 211 helplines across the USA, similar projects could be easily implemented with good prospects for sustainability. Third, methodologically, our study is among the first to evaluate the implementation of an adapted risk assessment screener for diseases of despair-related behaviors outside the context of a healthcare delivery setting. Our approach has the potential to reach a large number of underserved adults who might not be accessible through the healthcare system.

There were limitations to the present study. Our work was conducted in one region of Pennsylvania, so generalizability to other areas or states will be limited if our sample of callers differs significantly from those served by other 211 systems. Another limitation was that engagement data was self-reported by participants which brings the possibility of socially desirable answering. An effort was made to control for this by ensuring anonymity in survey activities. Also, given the limited scope of this feasibility study, it was not possible to track the outcomes of all referrals delivered to callers. Better data exchange coordination between helpline systems and those local partners providing social and health services is needed to better measure the impact of referrals on the disease of despair-related behaviors, uptake of flu vaccination, and long-term health outcomes. Another limitation was the small sample size of the survey study, which does not allow us to make definitive conclusions about callers’ intent to seek referral, their actual engagement with, or their satisfaction with resource navigators conducting the screener. However, the limited data collected in the present study suggests a positive experience among participants. Finally, to test some associations between screener results, referrals provided, and key demographic variables, we applied the Fisher’s exact test because the sample was very small (cells with < 5 observations). Our results must be interpreted considering this limitation. To address this limitation, future studies should enroll large samples of veterans and adults ages 65 or older in order to better assess these associations and confirm the results obtained in the present study.

In conclusion, our feasibility project shows that the 211 helpline is an ideal setting to identify and refer at-risk individuals to needed behavioral health care in the communities they live. With operations 24 hours a day, 7 days a week, and a highly qualified workforce, 211 helplines across the country can play a larger role in reaching out to vulnerable segments of the population and connect them with evidence-based programs to address diseases of despair-related behaviors.

## Supporting information

Calo et al. supplementary material 1 Calo et al. supplementary material

Calo et al. supplementary material 2 Calo et al. supplementary material

## Supplementary material

The supplementary material for this article can be found at https://doi.org/10.1017/cts.2024.548.
