# Supplementary figures and images for: Identifying at-risk individuals for diseases of despair through integration of clinical practice and social service systems
Source: J Clin Transl Sci. 2024 May 22;8(1):e100. doi: 10.1017/cts.2024.548 (PMC11639100; doi:10.1017/cts.2024.548)

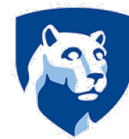

## IS THE CALLER ELIGIBLE TO PARTICIPATE IN THE PENN STATE STUDY?

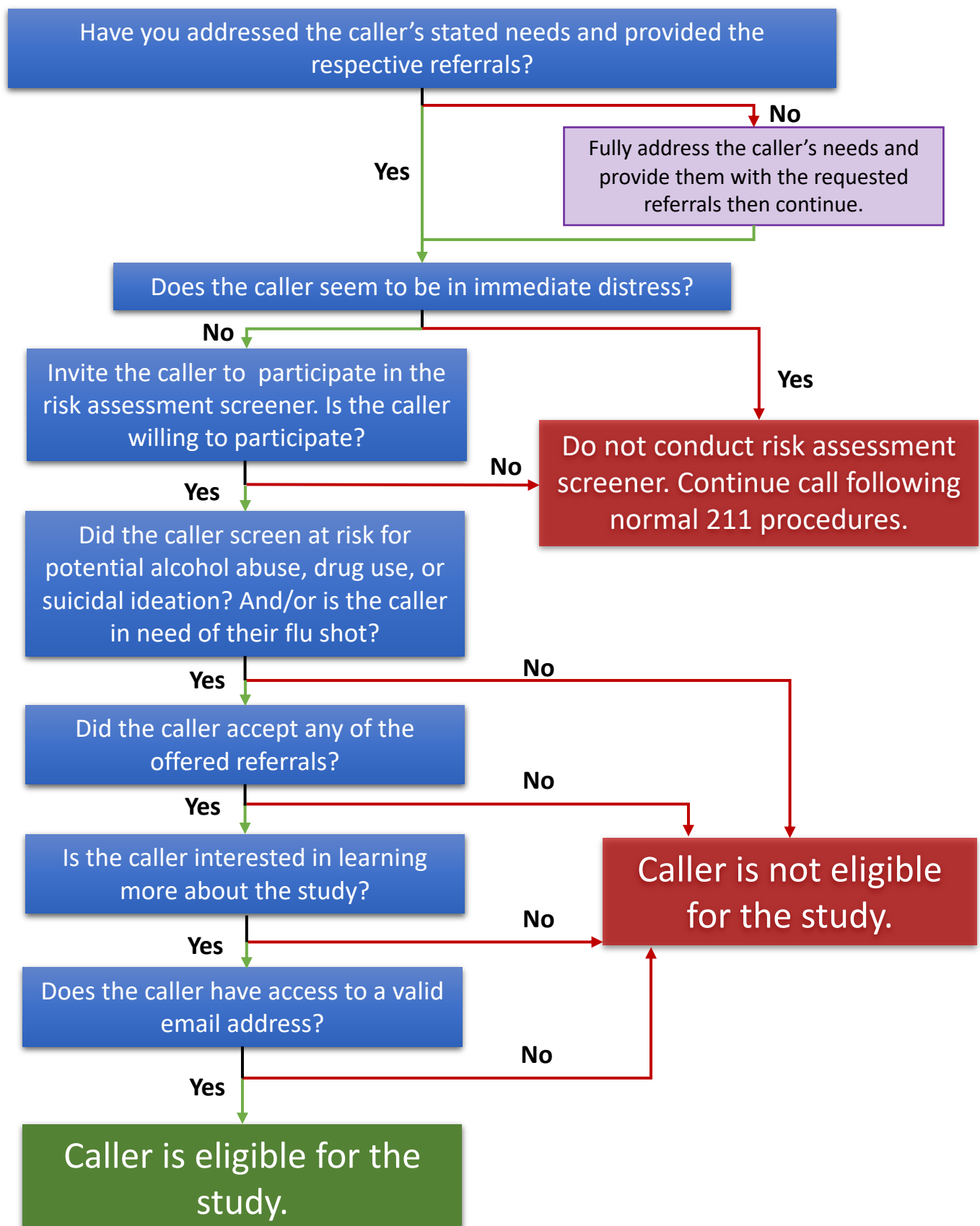

Supplement: Calo et al. supplementary material 1 — Calo et al. supplementary material [file S205986612400548Xsup001.pdf]
