# Supplementary material for: Identifying at-risk individuals for diseases of despair through integration of clinical practice and social service systems
Source: J Clin Transl Sci. 2024 May 22;8(1):e100. doi: 10.1017/cts.2024.548 (PMC11639100; doi:10.1017/cts.2024.548)
Supplement: Calo et al. supplementary material 2 — Calo et al. supplementary material [file S205986612400548Xsup002.docx]

**BASELINE SURVEY**

| **Item #** | **Variable Name** | **Item** | **Response scale** | **Source** |
| --- | --- | --- | --- | --- |
| **A. DEMOGRAPHICS** | | | | |
| A1 | Race | What is your race or ethnicity?  Check all that apply | 1, White  2, Black or African American  3, Asian  4, Native Hawaiian or Pacific Islander  5, American Indian or Alaska Native  6, Hispanic  7, Other | From HPV Qualtrics |
| A2 | Sex | What is your sex? | 1, Male  2, Female  3, Other | Adapted from BRFSS 2019 |
| A3 | Employment | Indicate your employment status. | 1, Employed full time  2, Employed part time  3, Unemployed  4, Unable to work or retired | Adapted from Kreuter et al |
| A4 | Dependents | How many people live in your household, including you? | Open-ended, range 1-20 | New item |
| A5 | Insurance | What health insurance do you have?  Check all that apply. | 1, Insurance through a current or former employer or union, including a spouse or partner’s employer  2, Medicare, Medicaid, or Medical Assistance  3, Tricare or other military health insurance  4, Other  5, Uninsured  **[5 = NONE OF THE ABOVE]** | Adapted from HINTS (J Moss survey) |
| A6 | Income | Indicate your level of household annual income. | 1, $15,000 or less  2, $15,000 - $24,999  3, $25,000 - $34,999  4, $35,000 - $49,999  5, $50,000 - $74,999  6, $75,000 or more | Adapted from Kreuter et al |
| A7 | Perceived health | Overall, how confident are you about your ability to take good care of your health? | 1, Completely confident  2, Very confident  3, Somewhat confident  4, A little confident  5, Not confident at all | HINTS (J Moss survey) |
| A8 | Preventive care provider | Not including mental health professionals, is there a particular doctor or other health professional that you see when you are sick or need advice about your health? | 1, No  2, Yes | HINTS (J Moss survey) |
| A9 | Preventive care visits | About how long has it been since you last visited a doctor for a routine checkup or a general physical exam? | 1, Within the past year  2, 1-2 years ago  3, 2-5 years ago  4, More than 5 years ago  5, Never | Adapted from HINTS (J Moss survey) |
| **B. SATISFACTION WITH 211 HELPLINE** | | | | |
|  | Prompt | The next questions are about the 211 helpline resource navigator you spoke to during the call where you agreed to participate in this study. |  |  |
| B1 | Ease of interaction | Say how much you agree or disagree with the next statements.  The resource navigator was easy to interact with. | 1, Strongly disagree  2, Somewhat disagree  3, Neither disagree or agree  4, Somewhat agree  5, Strongly agree | New item |
| B2 | Trustworthy | The resource navigator was trustworthy. | 1, Strongly disagree  2, Somewhat disagree  3, Neither disagree or agree  4, Somewhat agree  5, Strongly agree | New item |
| B3 | Knowledgeable | The resource navigator was knowledgeable. | 1, Strongly disagree  2, Somewhat disagree  3, Neither disagree or agree  4, Somewhat agree  5, Strongly agree | New item |
| B4 | Respectfulness | The resource navigator was respectful. | 1, Strongly disagree  2, Somewhat disagree  3, Neither disagree or agree  4, Somewhat agree  5, Strongly agree | New item |
| B5 | Helpful | The resource navigator was helpful. | 1, Strongly disagree  2, Somewhat disagree  3, Neither disagree or agree  4, Somewhat agree  5, Strongly agree | New item |
| B6 | Satisfaction | I would recommend others call the 211 to get referrals for health services. | 1, Strongly disagree  2, Somewhat disagree  3, Neither disagree or agree  4, Somewhat agree  5, Strongly agree | New item |
| **C. REFERRAL ASSESSMENT** | | | | |
| C1 | Referral type | In addition to the original reason you called the 211 helpline, what health service referrals were you given by the resource navigator?  Check all that apply. | 1, Alcohol use  2, Drug use or prescription medication overuse  3, Mental health or suicide prevention  4. Flu vaccination | New item |
|  | Prompt | Our records indicate that you received a referral for **[REFERRALS_PROVIDED]** |  |  |
| C2 | Referral intention ddap | What are your plans for seeking the alcohol and/or drug referral in the next month?  **[referrals_provided(1) = 1]** | 1, I plan to seek the referral  4, I was transferred by the 211 helpline resource navigator  2, I don’t plan to seek the referral  3, I am unsure | New item |
| C3 | Seek referral reason ddap | For which of the following reasons are you planning to seek the alcohol and/or drug referral?  Check all that apply.  **[ONLY IF C2 = 1]** | 1, It is important for my health or wellbeing  2, Needing this service  3, It was recommended by 211 Helpline resource navigator  4, Easy to seek  5, No or low cost to me  6, Convenient (e.g., service hours, location)  7, Have time for it  8, Have transportation to it  9, Supported by family or friends | New item |
| C4 | Not seek referral reason ddap | For which of the following reasons are you unsure or not planning to seek the alcohol and/or drug referral?  Check all that apply.  **[ONLY IF C2 = 2 or 3]** | 1, It is not important for my health or wellbeing  2, Not needing service  3, Embarrassment  4, Uneasy to seek  5, I cannot afford it or lack of insurance  6, Not convenient (e.g., service hours, location)  7, Lack of time  8, Lack of transportation  9, Not having support from family or friends | New item |
| C5 | Referral intention suicide | What are your plans for seeking the suicide/crisis referral in the next month?  **[referrals_provided(2)=1]** | 1, I plan to seek the referral  4, I was transferred by the 211 helpline resource navigator to get the health referral  2, I don’t plan to seek the referral  3, I am unsure | New item |
| C6 | Seek referral reason suicide | For which of the following reasons are you planning to seek the suicide/crisis referral?  Check all that apply.  **[ONLY IF C5 = 1** | 1, It is important for my health or wellbeing  2, Needing this service  3, It was recommended by 211 Helpline resource navigator  4, Easy to seek  5, No or low cost to me  6, Convenient (e.g., service hours, location)  7, Have time for it  8, Have transportation to it  9, Supported by family or friends | New item |
| C7 | Not seek referral reason suicide | For which of the following reasons are you unsure or not planning to seek the suicide/crisis referral?  Check all that apply.  **[ONLY IF C5 = 2 OR 3]** | 1, It is not important for my health or wellbeing  2, Not needing service  3, Embarrassment  4, Uneasy to seek  5, I cannot afford it or lack of insurance  6, Not convenient (e.g., service hours, location)  7, Lack of time  8, Lack of transportation  9, Not having support from family or friends | New item |
| C8 | Flu vaccine referral intention | What are your plans for seeking the flu shot/vaccine referral in the next month?  **[referrals_provided(3)=1]** | 1, I plan to seek the referral  2, I don’t plan to seek the referral  3, I am unsure | New item |
| C9 | Flu vaccine seek referral reasons | For which of the following reasons are you planning to seek the flu shot/vaccine referral?  Check all that apply.  **[ONLY IF C8 = 1]** | 1, I think flu vaccine works well  2, I have been sick with flu before and don’t want to be sick with it again  3, I think flu vaccine is safe  4, It was recommended by the 211 Helpline resource navigator  5, I always get the flu vaccine  6, It is low cost or covered by insurance  7, I have the time to go get the flu vaccine  8, I think the flu is a serious illness  9, Supported by family or friends | New item |
| C10 | Flu vaccine not seek referral reasons | For which of the following reasons are you unsure or not planning to seek the flu shot/vaccine referral?  Check all that apply.  **[ONLY IF C8 = 2 or 3]** | 1, I do not think flu vaccine works very well  2, I never get the flu  3, I am concerned about potential side effects from the vaccine  4, I am concerned about getting flu from the vaccine  5, I am concerned about potential exposure to COVID-19 if I go out to get the flu shot  6, I never get the flu vaccine  7, I cannot afford it  8, I do not know where to get vaccinated  9, I do not have time  10, I do not think the flu is a serious illness  11, I do not like vaccines  12, Not having support from family and friends | Adapted from NFID 2020 Flu Survey |
| **D. MEDICAL HISTORY FOR DISEASES OF DESPAIR** | | | | |
|  |  | **[THIS SECTION = DOD ONLY]** |  |  |
|  | Prompt | The next questions are about your health. |  |  |
| D1 | Health condition diagnosis | Have you ever been diagnosed by a doctor or other health care professional with any of the following?  Check all that apply. | 1, Depression  2, Anxiety  3, Panic disorder  4, Posttraumatic stress disorder  5, Bipolar disorder  6, Alcohol use disorder  7, Drug use disorder  8, Obsessive compulsive disorder  9, Other mental health condition  10, None  **[10 = NONE OF THE ABOVE]** | Adapted from College-Aged Adults Survey Summary from Anxiety and Depression Association of America |
| D2 | Health condition treatment | Which of the following types of treatment have you ever received for this diagnosed health condition?  Check all that apply.  **[SHOW IF D1 does NOT =10]** | 1, Talk therapy or psychotherapy  3, Peer support (e.g., support group)  4, Coaching (e.g., personal life coaching)  5, Prescription medication  6, Complementary/alternative treatments (e.g., acupuncture, meditation, yoga)  7, Other  8, I have never received treatment  **[8 = NONE OF THE ABOVE]** | Adapted from College-Aged Adults Survey Summary from Anxiety and Depression Association of America |
|  | Prompt | The next questions are based on information you provided to the resource navigator about:  [positive_screen].  **[positive_screen(1)]=1 or [positive_screen(2)]=1 or [positive_screen(3)]=1** |  |  |
| D3 | Friendfamily_ concern_ddap | Has a relative or friend been concerned about =your alcohol and/or drug use?  **[positive_screen(1)]=1 or [positive_screen(2)]=1** | 1, No  2, Yes | Adapted from Audit 10 |
| D4 | Doctor_concern_ddap | Has a doctor or another health care professional been concerned about your alcohol and/or drug use?  **[positive_screen(1)]=1 or [positive_screen(2)]=1** | 1, No  2, Yes | Adapted from Audit 10 |
| D5 | Dod_treatment_ddap | Have you ever been involved in a treatment program specifically related to your alcohol and/or drug use?  **[positive_screen(1)]=1 or [positive_screen(2)]=1** | 1, No  2, Yes | Adapted from DAST 28 |
| D6 | Dod_potentialtreatment_ddap | How often do you think alcohol and/or drug use can be treated?  **[positive_screen(1)]=1 or [positive_screen(2)]=1** | 1, Always  2, Often  3, Sometimes  4, Rarely  5, Never | Adapted from College-Aged Adults Survey Summary from Anxiety and Depression Association of America |
| D7 | Friendfamily_concern_suic | Has a relative or friend been concerned about your suicidal ideation/intention?  **[positive_screen(3)]=1** | 1, No  2, Yes | New item |
| D8 | Doctor_concern_suic | Has a doctor or another health care professional been concerned about your suicidal ideation/intention?  **[positive_screen(3)]=1** | 1, No  2, Yes | New item |
| D9 | Dod_treatment_suic | Have you ever been involved in a treatment program specifically related to your suicidal ideation/intention?  **[positive_screen(3)]=1** | 1, No  2, Yes | New item |
| D10 | Dod_potentialtreatment_suic | How often do you think suicidal ideation/intention can be treated?  **[positive_screen(3)]=1** | 1, Always  2, Often  3, Sometimes  4, Rarely  5, Never | New item |
| **E. FLU VACCINATION** | | | | |
|  |  | The next questions are about this flu season and the COVID-19 pandemic. |  |  |
| E1 | Covid_worry | How worried are you that you will get COVID-19 (or if previously infected, worried that you will get it again)? | 1, Not at all worried  2, A little worried  3, Somewhat worried  4, Very worried  5, Extremely worried | From NACC COVID impact survey  (NIH repository of COVID-19 research tools) |
| E2 | Flu_worry | How worried are you about getting the flu? | 1, Not at all worried  2, A little worried  3, Somewhat worried  4, Very worried  5, Extremely worried | NFID 2020 Flu Survey |
| E3 | Covid_likeliness | Compared to other people your age, how likely are you to get infected with COVID-19? | 1, Very unlikely  2, Unlikely  3, Neither unlikely nor likely  4, Likely  5, Very likely | Adapted from CDC HINTS survey |
| E4 | Flu_likeliness | Compared to other people your age, how likely are you to get the flu? | 1, Very unlikely  2, Unlikely  3, Neither unlikely nor likely  4, Likely  5, Very likely | Adapted from CDC HINTS survey |
| E5 | Flu_complications | As far as you know, do you have any of these health conditions at the present time?  Check all that apply. | 1, Diabetes  2, Asthma  3, Heart disease  4, Kidney disease  5, Obesity or overweight  6, None | NFID 2020 Flu Survey |
| E6 | Trust_providers | In general, how much would you trust information about vaccines from…  A doctor or other health care professional? | 1, A lot  2, Some  3, A little  4, Not at all | Adapted from CDC HINTS survey |
| E7 | Trust_familyfriends | Family or friends? | 1, A lot  2, Some  3, A little  4, Not at all | Adapted from CDC HINTS survey |
| E8 | Trust_211 | A resource navigator from the 211 helpline? | 1, A lot  2, Some  3, A little  4, Not at all | Adapted from CDC HINTS survey |
| E9 | Trust_socialmedia | Social media like Facebook, Twitter, or YouTube? | 1, A lot  2, Some  3, A little  4, Not at all | Adapted from CDC HINTS survey |
| E10 | Trust_govt | Government agencies like the CDC or state health departments? | 1, A lot  2, Some  3, A little  4, Not at all | Adapted from CDC HINTS survey |
| E11 | Flu_prevention | Say how much you agree or disagree with the next statements.  Flu vaccination is the best preventive measure against flu-related deaths and hospitalizations. | 1, Strongly disagree  2, Somewhat disagree  3, Neither disagree or agree  4, Somewhat agree  5, Strongly agree | NFID 2020 Flu Survey |
| E12 | Rn_infoproviders | Resource navigators from the 211 helpline are well-trained to provide information about the flu shot. | 1, Strongly disagree  2, Somewhat disagree  3, Neither disagree or agree  4, Somewhat agree  5, Strongly agree | NFID 2020 Flu Survey |
| **F. COVID-19 IMPACT** | | | | |
|  |  | The next questions ask about the impact you have experienced from the start of the COVID-19 pandemic until now. |  |  |
| F1 | Testing | Have you been tested for COVID-19? | 1, Yes, I was tested, and the test was positive.  2, Yes, I was tested, and the test was negative.  3, Yes, I was tested, and am waiting on the results.  4, No, I tried to get tested but could not get one.  5, No, I have not tried to get tested. | Stanford COVID-19 on patients with cancer testing  (NIH repository of COVID-19 research tools) |
| F2 | Impact_everyday | How disruptive has the COVID-19 pandemic been to your everyday life? | 1, Not at all disruptive  2, A little disruptive  3, Somewhat disruptive  4, Very disruptive  5, Extremely disruptive | NACC  (NIH repository of COVID-19 research tools) |
| F3 | Impact_income | Has your household’s income been significantly reduced due to COVID-19? | 1, No  2, Yes | NACC  (NIH repository of COVID-19 research tools) |
| F4 | Impact_mentalhealth | Has your mental health gotten better or worse since the start of the pandemic? | 1, Yes, gotten better  2, Yes, gotten worse  3, No, there has been no change | Vanderbilt Child Health COVID-19 poll  (NIH repository of COVID-19 research tools) |
| F5 | Covid_alcohol | Since the start of the COVID-19 pandemic, you drink alcohol (beer, wine, liquor, etc)… | 1, Less often  2, About the same  3, More often  4, I don’t drink alcohol | CPTS  (NIH repository of COVID-19 research tools) |
| F6 | Covid_drugs | Since the start of the COVID-19 pandemic, you use drugs… | 1, Less often  2, About the same  3, More often  4, I don’t use drugs | Adapted from CPTS  (NIH repository of COVID-19 research tools) |
| F7 | Covid_211info | How likely are you to use the 211 Helpline to get information on COVID-19? | 1, Very unlikely  2, Unlikely  3, Neither unlikely nor likely  4, Likely  5, Very likely | New item |
| F8 | Covid_211testing | How likely are you to use the 211 Helpline to get information on COVID-19 testing? | 1, Very unlikely  2, Unlikely  3, Neither unlikely nor likely  4, Likely  5, Very likely | New item |
| F9 | Covid_211vax | How likely would you be to use the 211 Helpline to get information on obtaining a COVID-19 vaccine once available? | 1, Very unlikely  2, Unlikely  3, Neither unlikely nor likely  4, Likely  5, Very likely | New item |
| **G. COVID-19 VACCINATION** | | | | |
| G1 | Covidvax_intentions | When a vaccine for COVID-19 becomes available, will you get vaccinated? | 1, Yes  2, No  3, Not sure | AmeriSpeak survey |
| G2 | Covidvax_setting | In which settings would you be willing to get a potential COVID-19 vaccine?  Check all that apply. | 1, Doctor’s office  2, Pharmacy  3, Health fair or community event  4, Local health department  5, Urgent care clinic  6, Emergency room  7, Drive-through or walk-up clinics  8, Other  9, I would not be willing to get it  **[9 = NONE OF THE ABOVE]** | Adapted from HPV qualtrics |
| G3 | Covidvax_concern | Imagine that you wanted more information on a COVID-19 vaccine available to you.  Would you like information about…  Check all that apply. | 1, Safety or side effects  2, Efficacy  3, Cost  4, Number of doses  5, Places to get the vaccine  6, Other information | Adapted from HPV qualtrics |
| G4 | Covidinfo_source | From which sources would you prefer to receive this information?  Check all that apply. | 1, Government agencies like the CDC or state health departments  2, Doctor or other health care professionals  3, United Way 211 Helpline  4, Internet or social media  5, Family or friends  6, Other | New item |
| **H. SOCIAL FACTORS** | | | | |
|  |  | The next questions ask about your relationships with the important people in your life |  |  |
| H1 | Socialsupport_doctor | Since the start of the pandemic, how often would you have…  Someone to take you to the doctor if you need it? | 1, None of the time  2, A little of the time  3, Some of the time  4, Most of the time  5, All of the time | mMOS-SS 8 (From J Moss Survey) |
| H2 | Socialsupport_suggestions | Someone to turn to for suggestions about how to deal with a personal problem? | 1, None of the time  2, A little of the time  3, Some of the time  4, Most of the time  5, All of the time | mMOS-SS 8 (From J Moss Survey) |
| H3 | Socialsupport_listen | Someone to listen to you? | 1, None of the time  2, A little of the time  3, Some of the time  4, Most of the time  5, All of the time | mMOS-SS 8 (From J Moss Survey) |
| H4 | Socialsupport_info | Someone to give you health information? | 1, None of the time  2, A little of the time  3, Some of the time  4, Most of the time  5, All of the time | mMOS-SS 8 (From J Moss Survey) |

**FOLLOW-UP SURVEY**

| **Item #** | **Variable Name** | **Item** | **Response scale** | **Source** |
| --- | --- | --- | --- | --- |
| **A. REFERRAL UTILIZATION** | | | | |
|  | Prompt_post | Our records indicate that you received referral(s) for **[referrals_provided]** around a month ago. |  |  |
| A1 | Referral_engage_level_ddap | What best describes your status with the alcohol and/or drug use referral?  **[referrals_provided(1)]=1** | 1, Searched for more information about it  2, Called and spoke with someone  3, Called, but nobody answered  4, Scheduled an appointment  5, Attended referral  6, I was transferred by the 211 Helpline resource navigator to get the health referral  7, I did not seek the referral | New item |
| A2 | dod_no_engage_ddap | What was your reason for not engaging with the alcohol and/or drug use referral?  Check all that apply.  **[ONLY IF A1 = 7]** | 1, It is not important for my health or wellbeing  2, Not needing service  3, Embarrassment  4, Uneasy to seek  5, I cannot afford it or lack of insurance  6, Not convenient (e.g., service hours, location)  7, Lack of time  8, Lack of transportation  9, Not having support from family or friends | New item |
| A3 | Dod_engagement_ddap | What was your reason for engaging with the alcohol and/or drug use referral?  Check all that apply.  **[ONLY IF A1 = 1-6]** | 1, It is important for my health or wellbeing  2, Needing this service  3, It was recommended by the 211 Helpline resource navigator  4, Easy to seek  5, No or low cost to me  6, Convenient (e.g., service hours, location)  7, Had time for it  8, Had transportation to it  9, Supported by family or friends | New item |
| A4 | Referral_engage_level_suic | What best describes your status with the suicide/crisis referral?  **[referrals_provide(2)]=1** | 1, Searched for more information about it  2, Called and spoke with someone  3, Called, but nobody answered  4, Scheduled an appointment  5, Attended referral  6, I was transferred by the 211 Helpline resource navigator to get the health referral  7, I did not seek the referral | New item |
| A5 | Dod_no_engage_suic | What was your reason for not engaging with the suicide/crisis referral?  Check all that apply.  **[ONLY IF A4=7]** | 1, It is not important for my health or wellbeing  2, Not needing service  3, Embarrassment  4, Uneasy to seek  5, I cannot afford it or lack of insurance  6, Not convenient (e.g., service hours, location)  7, Lack of time  8, Lack of transportation  9, Not having support from family or friends | New item |
| A6 | Dod_engage_suic | What was your reason for engaging with the suicide/crisis referral?  Check all that apply.  **[ONLY IF A4=1-6]** | 1, It is important for my health or wellbeing  2, Needing this service  3, It was recommended by the 211 Helpline resource navigator  4, Easy to seek  5, No or low cost to me  6, Convenient (e.g., service hours, location)  7, Had time for it  8, Had transportation to it  9, Supported by family or friends | New item |
| A7 | Referral_engage_level_flu | What best describes your status with the flu shot/vaccine referral?  **[referrals_provided(3)]=1** | 1, Searched for more information about it  2, Called and spoke with someone  3, Called, but nobody answered  4, Scheduled an appointment  5, Attended referral  6, I was transferred by the 211 Helpline resource navigator to get the health referral  7, I did not seek the referral | New item |
| A8 | Flu_no_engagement | What was your reason for not engaging with this referral?  Check all that apply.  **[ONLY IF A7=7]** | 1, I do not think flu vaccine works very well  2, I never get the flu  3, I am concerned about potential side effects from the vaccine  4, I am concerned about getting flu from the vaccine  5, I am concerned about potential exposure to COVID-19 if I go out to get the flu shot  6, I never get the flu vaccine  7, I cannot afford it  8, I do not know where to get vaccinated  9, I do not have time  10, I do not think the flu is a serious illness  11, I do not like vaccines  12, Not having support from family and friends | Adapted from NFID 2020 Flu Survey |
| A9 | Flu_engagement | What was your reason for engaging with this referral?  Check all that apply.  **[ONLY IF A7=2-6]** | 1, I think flu vaccine works well  2, I have been sick with flu before and don’t want to be sick with it again  3, I think flu vaccine is safe  4, It was recommended by the 211 Helpline resource navigator  5, I always get the flu vaccine  6, It is low cost or covered by insurance  7, I have the time to go get the flu vaccine  8, I think the flu is a serious illness  9, Supported by family or friends | New item |
| A10 | Post_helpline_rec | Say how much you agree or disagree with the next statement.  I would recommend others to call the 211 to get referrals for health services. | 1, Strongly disagree  2, Somewhat disagree  3, Neither disagree or agree  4, Somewhat agree  5, Strongly agree | New item |
| **B. FLU VACCINATION** | | | | |
|  |  | The next questions are about this flu season and the COVID-19 pandemic. |  |  |
| B1 | Post_covid_worry | How worried are you that you will get COVID-19 (or if previously infected, worried that you will get it again)? | 1, Not at all worried  2, A little worried  3, Somewhat worried  4, Very worried  5, Extremely worried | From NACC COVID impact survey  (NIH repository of COVID-19 research tools) |
| B2 | Post_flu_worry | How worried are you about getting the flu? | 1, Not at all worried  2, A little worried  3, Somewhat worried  4, Very worried  5, Extremely worried | NFID 2020 Flu Survey |
| B3 | Post_covid_likeliness | Compared to other people your age, how likely are you to get infected with COVID-19? | 1, Very unlikely  2, Unlikely  3, Neither unlikely nor likely  4, Likely  5, Very likely | Adapted from CDC HINTS survey |
| B4 | Post_flu_likeliness | Compared to other people your age, how likely are you to get the flu? | 1, Very unlikely  2, Unlikely  3, Neither unlikely nor likely  4, Likely  5, Very likely | Adapted from CDC HINTS survey |
| B5 | Post_flu_complications | As far as you know, do you have any of these health conditions at the present time?  Check all that apply. | 1, Diabetes  2, Asthma  3, Heart disease  4, Kidney disease  5, Obesity or overweight  6, None | NFID 2020 Flu Survey |
| B6 | Post_trust_provider | In general, how much would you trust information about vaccines from…  A doctor or other health care professional? | 1, A lot  2, Some  3, A little  4, Not at all | Adapted from CDC HINTS survey |
| B7 | Post_trust_familyfriends | Family or friends? | 1, A lot  2, Some  3, A little  4, Not at all | Adapted from CDC HINTS survey |
| B8 | Post_trust_211 | A resource navigator from the 211 helpline? | 1, A lot  2, Some  3, A little  4, Not at all | Adapted from CDC HINTS survey |
| B9 | Post_trust_socialmedia | Social media like Facebook, Twitter, or YouTube? | 1, A lot  2, Some  3, A little  4, Not at all | Adapted from CDC HINTS survey |
| B10 | Post_Trust_govt | Government agencies like the CDC or state health departments? | 1, A lot  2, Some  3, A little  4, Not at all | Adapted from CDC HINTS survey |
| B11 | Post_flu_prevention | Say how much you agree or disagree with the next statements.  Flu vaccination is the best preventive measure against flu-related deaths and hospitalizations. | 1, Strongly disagree  2, Somewhat disagree  3, Neither disagree or agree  4, Somewhat agree  5, Strongly agree | NFID 2020 Flu Survey |
| B12 | Post_rn_infoproviders | Resource navigators from the 211 helpline are well-trained to provide information about the flu shot. | 1, Strongly disagree  2, Somewhat disagree  3, Neither disagree or agree  4, Somewhat agree  5, Strongly agree | NFID 2020 Flu Survey |
| **C. COVID-19 IMPACT** | | | | |
|  |  | The next questions ask about the impact you have experienced from the start of the COVID-19 pandemic until now. |  |  |
| C1 | Post_testing | Have you been tested for COVID-19? | 1, Yes, I was tested, and the test was positive.  2, Yes, I was tested, and the test was negative.  3, Yes, I was tested, and am waiting on the results.  4, No, I tried to get tested but could not get one.  5, No, I have not tried to get tested. | Stanford COVID-19 on patients with cancer testing  (NIH repository of COVID-19 research tools) |
| C2 | Post_impact_everyday | How disruptive has the COVID-19 pandemic been to your everyday life? | 1, Not at all disruptive  2, A little disruptive  3, Somewhat disruptive  4, Very disruptive  5, Extremely disruptive | NACC  (NIH repository of COVID-19 research tools) |
| C3 | Post_impact_income | Has your household’s income been significantly reduced due to COVID-19? | 1, No  2, Yes | NACC  (NIH repository of COVID-19 research tools) |
| C4 | Post_impact_mentalhealth | Has your mental health gotten better or worse since the start of the pandemic? | 1, Yes, gotten better  2, Yes, gotten worse  3, No, there has been no change | Vanderbilt Child Health COVID-19 poll  (NIH repository of COVID-19 research tools) |
| C5 | Post_covid_alcohol | Since the start of the COVID-19 pandemic, you drink alcohol (beer, wine, liquor, etc)… | 1, Less often  2, About the same  3, More often  4, I don’t drink alcohol | CPTS  (NIH repository of COVID-19 research tools) |
| C6 | Post_covid_drugs | Since the start of the COVID-19 pandemic, you use drugs… | 1, Less often  2, About the same  3, More often  4, I don’t use drugs | Adapted from CPTS  (NIH repository of COVID-19 research tools) |
| C7 | Post_covid_211info | How likely are you to use the 211 Helpline to get information on COVID-19? | 1, Very unlikely  2, Unlikely  3, Neither unlikely nor likely  4, Likely  5, Very likely | New item |
| C8 | Post_covid_211testing | How likely are you to use the 211 Helpline to get information on COVID-19 testing? | 1, Very unlikely  2, Unlikely  3, Neither unlikely nor likely  4, Likely  5, Very likely | New item |
| C9 | Post_covid_211vax | How likely would you be to use the 211 Helpline to get information on obtaining a COVID-19 vaccine once available? | 1, Very unlikely  2, Unlikely  3, Neither unlikely nor likely  4, Likely  5, Very likely | New item |
| **D. COVID-19 VACCINATION** | | | | |
| D1 | Post_covidvax_intentions | When a vaccine for COVID-19 becomes available, will you get vaccinated? | 1, Yes  2, No  3, Not sure | AmeriSpeak survey |
| D2 | Post_covidvax_setting | In which settings would you be willing to get a potential COVID-19 vaccine?  Check all that apply. | 1, Doctor’s office  2, Pharmacy  3, Health fair or community event  4, Local health department  5, Urgent care clinic  6, Emergency room  7, Drive-through or walk-up clinics  8, Other  9, I would not be willing to get it  **[9 = NONE OF THE ABOVE]** | Adapted from HPV qualtrics |
| D3 | Post_covidvax_concern | Imagine that you wanted more information on a COVID-19 vaccine available to you.  Would you like information about…  Check all that apply. | 1, Safety or side effects  2, Efficacy  3, Cost  4, Number of doses  5, Places to get the vaccine  6, Other information | Adapted from HPV qualtrics |
| D4 | Post_covidinfo_source | From which sources would you prefer to receive this information?  Check all that apply. | 1, Government agencies like the CDC or state health departments  2, Doctor or other health care professionals  3, United Way 211 Helpline  4, Internet or social media  5, Family or friends  6, Other | New item |
